# Ru^III^(edta) complexes as molecular redox catalysts in chemical and electrochemical reduction of dioxygen and hydrogen peroxide: inner-sphere *versus* outer-sphere mechanism†

**DOI:** 10.1039/d1ra03293c

**Published:** 2021-06-16

**Authors:** Debabrata Chatterjee, Marta Chrzanowska, Anna Katafias, Maria Oszajca, Rudi van Eldik

**Affiliations:** Vice-Chancellor's Research Group at Zoology Department, University of Burdwan Burdwan-713104 India dchat57@hotmail.com; Faculty of Chemistry, Nicolaus Copernicus University in Toruń Gagarina 7 87-100 Toruń Poland rudi.vaneldik@fau.de; Faculty of Chemistry, Jagiellonian University Gronostajowa 2 30-387 Kraków Poland; Department of Chemistry and Pharmacy, University of Erlangen-Nuremberg Egerlandstr. 1 91058 Erlangen Germany

## Abstract

The reduction of molecular oxygen (O_2_) and hydrogen peroxide (H_2_O_2_) by [Ru^II^(edta)(pz)]^2−^ (edta^4−^ = ethylenediaminetetraacetate; pz = pyrazine) has been studied spectrophotometrically and kinetically in aqueous solution. Exposure of the aqua-analogue [Ru^II^(edta)(H_2_O)]^2−^ to O_2_ and H_2_O_2_ resulted in the formation of [Ru^III^(edta)(H_2_O)]^−^ species, with subsequent formation of the corresponding Ru^V^=O complex. A working mechanism for the O_2_ and H_2_O_2_ reduction reactions mediated by the Ru^II^(edta) complexes is proposed. The role of the coordinated water molecule (by its absence or presence in the primary coordination sphere) in controlling the mechanistic pathways, outer-sphere or inner-sphere, is discussed.

## Abstract 2

[Ru^II^(edta)(L)]^2–^, where edta^4–^ =ethylenediaminetetraacetate; L = pyrazine (pz) and H_2_O, can reduce molecular oxygen sequentially to hydrogen peroxide and further to water by involving both outer-sphere and inner-sphere electron transfer processes.

## Graphical abstract

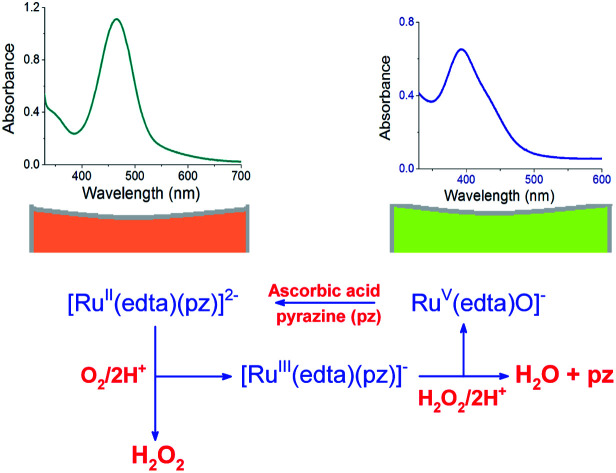

## Introduction

**Scheme 1:**
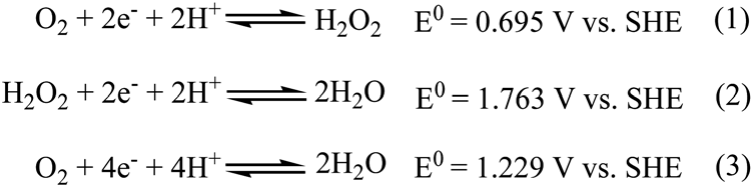
Pictorial presentation of proton-coupled electron transfer reactions for dioxygen reduction. SHE = Standard Hydrogen Electrode.

The electrochemical oxygen reduction reaction (ORR) proceeds by two-electron two-proton (2e^−^/2H^+^) partial reduction of O_2_ to produce H_2_O_2_ (eqn (1) in Scheme 1) or direct four-electron four-proton (4e^−^/4H^+^) reduction of O_2_ to 2H_2_O (eqn (3) in Scheme 1). Although selective reduction of O_2_ directly to H_2_O is of continued interest in regard to its application in energy conversion, particularly in the field of fuel cells and metal–air batteries,^1–4^ production of H_2_O_2_*via* two-electron two-proton reduction of O_2_ is also of considerable importance for environmental application like waste water treatment and chemical feedstocks.^5,6^

Use of transition metal complexes as molecular catalysts as a redox mediator to affect the reduction of oxygen is well documented in the literature.^7–12^ Many schemes of catalytic processes that affect reduction of O_2_ in combination of 2e^−^/2H^+^ and/or 4e^−^/4H^+^ pathways, either selectively or sequentially, have been reported. Noteworthy here, is that mechanistic details and kinetic parameters that control the efficiency of the four-electron reduction of O_2_ to H_2_O *versus* two-electron partial reduction of O_2_ to H_2_O_2_ are still lacking, even though exhaustive efforts have been devoted for more than the last two-decades in mimicking and understanding the enzymatic activity of *cytochrome c* oxidase (which catalyses the direct four electron reduction of O_2_ to H_2_O during the final stage of respiration^13^). Another important aspect is, that although the reduction of hydrogen peroxide to water (eqn (2)) is seemingly easier thermodynamically than the reduction of dioxygen to hydrogen peroxide (eqn (1)), it is kinetically very difficult as it involves the cleavage of the O–O bond.

While mononuclear as well as binuclear complexes of copper, iron, cobalt and manganese, have been exhaustively studied pertaining to the oxygen reduction reaction,^7–12^ the use of the ruthenium complex in this context is scanty in the literature.^14^ In the present work, we set out to examine the ability of the Ru^II^(edta) complex (edta^4−^ = ethylenediaminetetraacetate) to affect the reduction reaction of molecular oxygen in aqueous medium. The feature that dominates the chemistry of the [Ru^III^(edta)(H_2_O)]^−^ complex is its lability towards aqua-substitution reactions,^15^ which affords an advantage of facile and straightforward binding of substrate molecules to the metal centre. In addition, a range of accessible and stable oxidation states made Ru(edta) complexes abidingly important to the catalytic studies for the past two decades. The significance of the Ru(edta) complexes in mimicking enzymatic redox reactions and small molecule activation, have been well established in very recent review articles.^16,17^

We for the first time explore, that [Ru^II^(edta)(pz)]^2−^ (pz = pyrazine) and its aqua-analogue [Ru^II^(edta)(H_2_O)]^2−^, can efficiently mediate the sequential 2e^−^/2H^+^ reduction of O_2_ to H_2_O_2_ and further reduction of H_2_O_2_ to H_2_O. We report herein the results of the detailed spectral and kinetic investigation of the reduction of O_2_ and H_2_O_2_ by the above referred Ru^II^(edta) complexes.

## Experimental

### Materials

The starting complex K[Ru^III^(Hedta)Cl]·2H_2_O was prepared and characterized as described elsewhere.^18^ The complex K[Ru^III^(Hedta)Cl] instantaneously converts into [Ru^III^(Hedta)(H_2_O)] when dissolved in water.^19,20^ The p*K*_a_ values associated with the acid-dissociation equilibria of the pendant carboxylic acid arm and the coordinated water molecule, are 2.4 and 7.6, respectively, at 25 °C.^19,20^ All other reagents and buffer components used were of the highest grade commercially available and were used as received. Doubly distilled H_2_O was used to prepare all solutions.

### Instrumentation

The reactions were studied applying UV-Vis spectroscopy. Fast kinetic measurements were performed using a stopped-flow (Applied Photophysics SX20) equipped with a rapid-scan diode-array spectrometer with a multi-wavelength J&M detector. The solution temperature was maintained at the desired temperature within ±0.1 °C using a Colora thermostat. The time courses of the slow reactions were followed spectrophotometrically, adopting a conventional mixing technique using the HP 8453 diode-array spectrophotometer. A tandem cuvette was used for this purpose. This instrument was thermostated at the desired temperature (±0.1 °C) using a HP 89090 Peltier Temperature Controller. Since, Ru^II^(edta) complexes are very air sensitive, all experimental solutions of the Ru^II^-complexes were prepared strictly under Ar atmosphere. Gas-tight Hamilton syringes were used to transfer these solutions throughout the studies. Preparation of oxygen saturated solutions was accomplished by bubbling pure oxygen through the deaerated double-distilled water for 30 min. This solution was carefully diluted by mixing with deaerated water in desired proportion. The pH of the buffer solution was measured with an Elmetron CP-505 pH meter. Acetate buffer (0.1 M) was used to control the pH of the experimental solutions. Several kinetic runs (at least three) were performed to obtain reproducible results within ±4%.

## Results and discussion

The ‘edta’ ligand functions as a pentadentate ligand towards Ru(iii) with a pendant acetate arm.^21^ The [Ru^III^(edta)H_2_O]^−^ complex reacts with pyrazine (pz) to form the [Ru^III^(edta)(pz)]^−^ complex, through a rapid and straightforward water displacement reaction (*k*_f_ = 2 × 10^4^ M^−1^ s^−1^ and *k*_r_ = 2 s^−1^ at 25 °C)^19^ as outlined in eqn (4).4
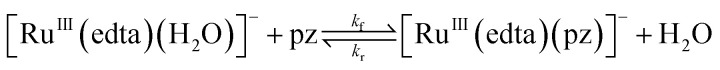


**Fig. 1:**
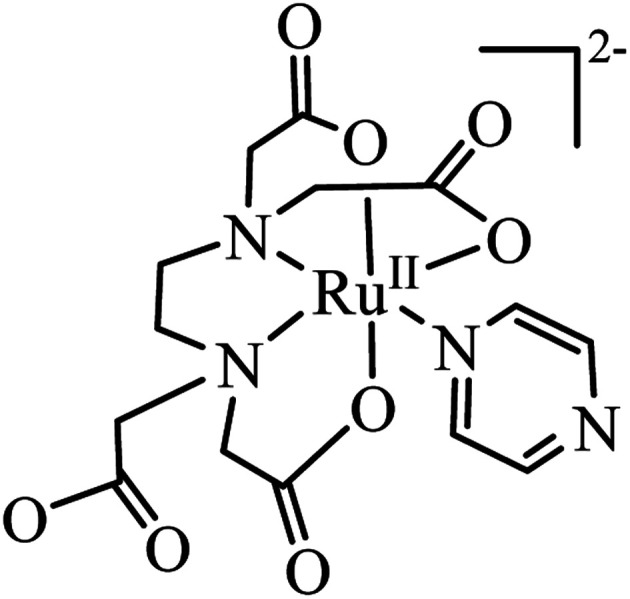
Pictorial presentation of [Ru^II^(edta)(pz)]^2−^.

The [Ru^III^(edta)(pz)]^−^ complex can easily be reduced electrochemically (*E*_1/2_ value corresponding to the [Ru^III^(edta)(pz)]^−^/[Ru^II^(edta)(pz)]^2−^ couple is 0.252 V *vs.* SHE)^19^ or chemically (in the presence of reducing agents *viz.* ascorbic acid)^22^ to its ruthenium(ii) analogue, [Ru^II^(edta)(pz)]^2−^ (Fig. 1). The facile inter-conversion between low-spin Ru^II^/Ru^III^ redox states, does not require significant needs for structural changes that limit electron transfer steps.

The electronic absorption spectrum of the [Ru^III^(edta)(pz)]^−^ complex in aqueous solution is featureless in the entire visible range, whereas its Ru(ii)-analogue, [Ru^II^(edta)(pz)]^2−^ (Fig. 1) displays a strong band in the visible range (*λ*_max_ = 462 nm, *ε*_max_ = 11 600 M^−1^ cm^−1^) which was assigned to a metal to ligand charge transfer (MLCT) band.^19^ This huge spectral difference thus offers an amenable way to follow the kinetics of the electron transfer reactions spectrophotometrically.

### Reduction of O_2_ by [Ru^II^(edta)(pz)]^2−^

Addition of an oxygen-saturated aqueous solution to the deaerated red solution of the [Ru^II^(edta)(pz)]^2−^ complex (see S1 in ESI†) at pH 5.0 (acetate buffer), resulted in a gradual disappearance of the red colour.

**Fig. 2:**
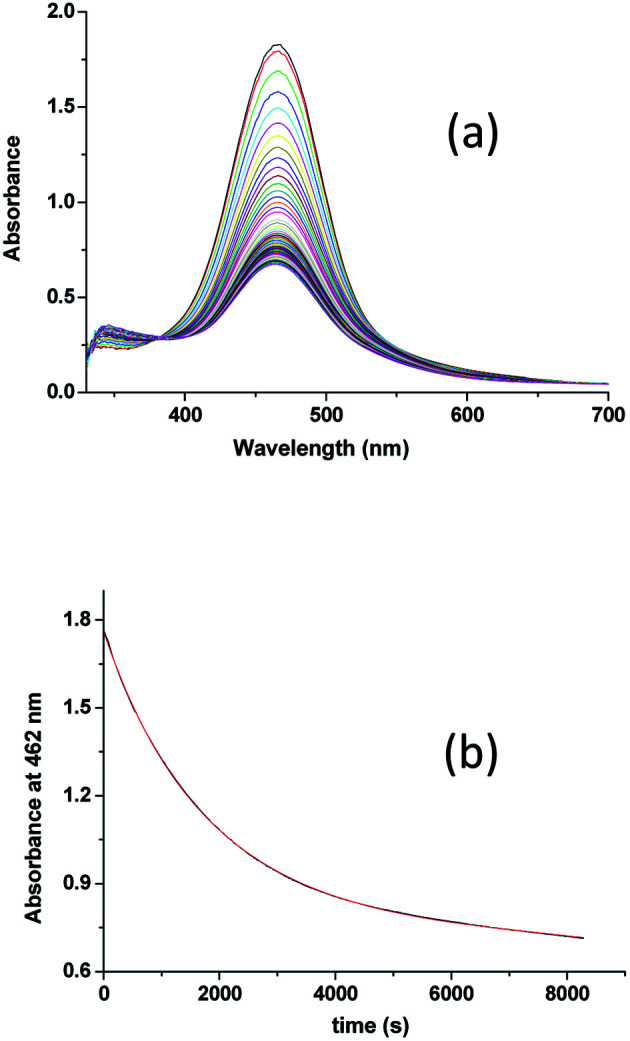
(a) Spectral changes that occurred during oxidation of [Ru^II^(edta)(pz)]^2−^ with O_2_ at 25 °C and pH 5.0 and (b) absorbance *vs.* time trace recorded at 462 nm, [Ru] = 0.25 mM, [O_2_] = 0.5 mM.

The overall spectral changes recorded immediately after mixing the solutions of [Ru^II^(edta)(pz)]^2−^ and aqueous solution of dissolved oxygen, are shown in Fig. 2a. The spectral changes are attributed to the oxidation of [Ru^II^(edta)(pz)]^2−^ to [Ru^III^(edta)(pz)]^−^, and a typical kinetic trace recorded at 462 nm (decay) is shown in Fig. 2b.

**Scheme 2:**
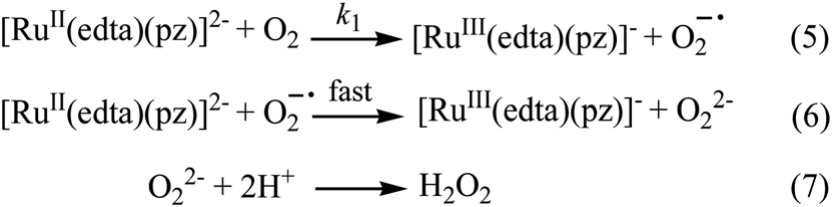
Proposed mechanism for the reduction of O_2_ by [Ru^II^(edta)(pz)]^2−^.

Effect of the concentration of the dissolved oxygen on the rate of the reaction was studied at 25 °C and pH 5.0 (representative kinetic traces recorded at 462 nm are shown in Fig. S1 in ESI†). Under the specified conditions, the rate of the reaction estimated by the maximum slope, increases linearly with increasing concentration of dissolved oxygen (Fig. S2 in ESI†). The spectral and kinetic observations can be accounted for in terms of the reaction sequence outlined in Scheme 2. The rate-determining step (5) proposed in the mechanism, involves a one-electron transfer from [Ru^II^(edta)(pz)]^2−^ to the O_2_ to yield O_2_^−^˙ radical species in an outer-sphere manner. In the subsequent and kinetically inconsequential step (eqn (6)), the O_2_^−^˙ radical rapidly reacts with another molecule of [Ru^II^(edta)(pz)]^2−^ to produce the peroxide ion (O_2_^2−^), protonation of which (eqn (7)) results in the formation of hydrogen peroxide (H_2_O_2_).

The reduction of O_2_ with [Ru^II^(edta)(pz)]^2−^ under the specified conditions can be accounted for in terms of the rate-law expressed by eqn (8).

8 Rate = *k*_1_[Ru^II^(edta)(pz)^2−^][O_2_]

The value of the second-order rate constant (*k*_1_) estimated from the slope of the plot of rate *versus* [O_2_] shown in Fig. S2 (in ESI†) is 0.14 ± 0.01 M^−1^ s^−1^ at 25 °C. Addition of fresh ascorbic acid to the resultant solution obtained at the end of the aforementioned reaction (experimental conditions given under Fig. 2), resulted in the formation of the [Ru^II^(edta)(pz)]^2−^ almost quantitatively as evidenced by the spectral measurements (Fig. S3 in ESI†). The above observations clearly indicate the existence of a catalytic process in the overall reactions, wherein dioxygen (O_2_) is reduced to hydrogen peroxide (H_2_O_2_) *via* an electron transfer reaction, and the [Ru^III^(edta)(pz)]^−^ complex acts as a redox relay for electron transmission from ascorbic acid to O_2_.

### Reduction of H_2_O_2_ by [Ru^II^(edta)(pz)]^2−^

In order to understand the reaction of [Ru^II^(edta)(pz)]^2−^ with H_2_O_2_ (formed during the reduction of O_2_ by [Ru^II^(edta)(pz)]^2−^ as shown in Scheme 2), we performed a detailed kinetic study of the reaction of [Ru^II^(edta)(pz)]^2−^ with H_2_O_2_ discretely under similar conditions of pH (5.0) and temperature (25 °C).

**Fig. 3:**
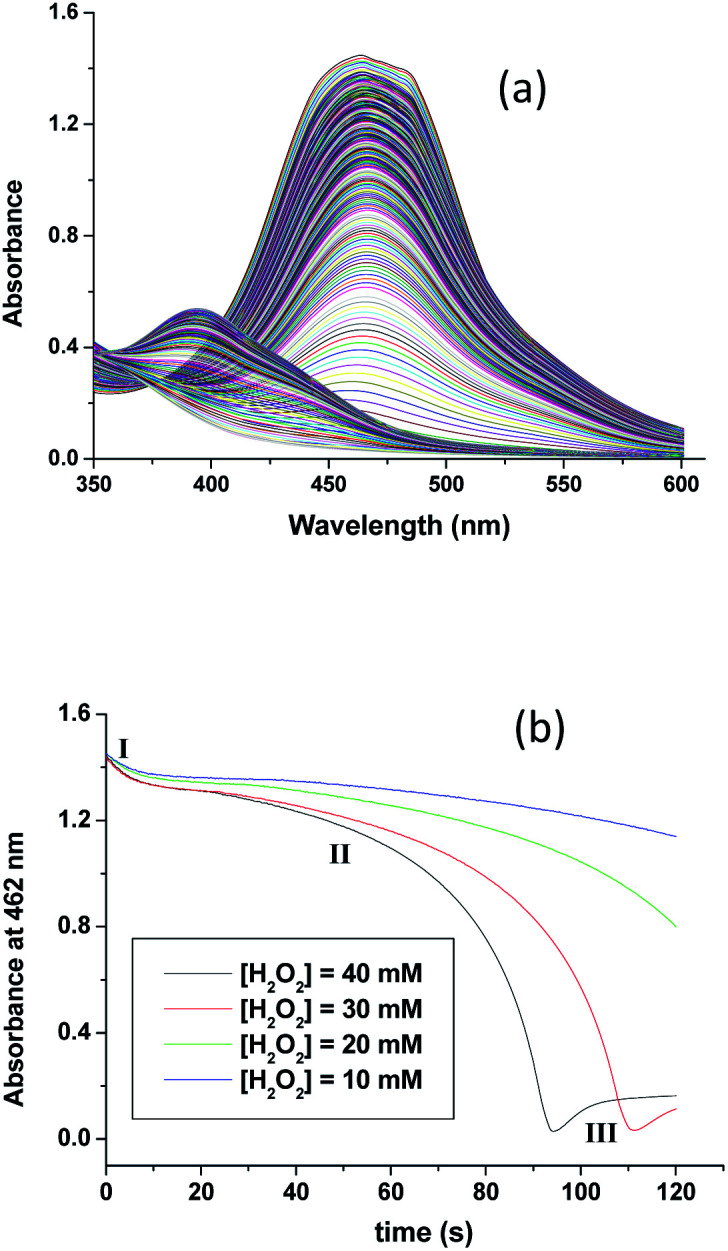
(a) Spectral changes that occurred during reaction of [Ru^II^(edta)(pz)]^2−^ with H_2_O_2_ and (b) absorbance *vs.* time traces as a function of [H_2_O_2_] at 25 °C and pH 5.0, [Ru] = 0.25 mM.

In Fig. 3a typical UV-visible spectral changes with time are shown (recorded by using stopped-flow rapid scan, diode array spectrophotometer) that occurred upon mixing an aqueous solution of [Ru^II^(edta)(pz)]^2−^ with the solution of H_2_O_2_ (in acetate buffer). The overall kinetic trace (Fig. 3b) derived from the time-resolved spectral changes, exhibited three clear steps (two decay and one growth at higher [H_2_O_2_]) marked as I, II and III, respectively). The first decay step involves a small decrease in absorbance, not of enough significance (less than 5% as compared to the total absorbance change in the overall reaction time). Kinetic traces (see Fig. S4 in ESI†) pertinent to step I analysed on a shorter time scale as a function of [H_2_O_2_], are seemingly exponential in nature and could be fitted with a single exponential function within the precision of experimental data (*R* > 0.99). The values of the observed rate constant (*k*_obs_ = 0.18 ± 0.02 s^−1^ at 25 °C and pH 5.0) so obtained were found to be independent of the H_2_O_2_ concentration. The spectral changes involved in step I may be attributed to the formation of a very weak [Ru^II^(edta)(Ac)]^3−^ (Ac = acetate) complex by the reaction of [Ru^II^(edta)(pz)]^2−^ with buffer component (acetate) for which the slight collapse of the MLCT band at 462 nm is seen due to removal of the coordinated N-heterocyclic ligand pyrazine (pz), in a [H_2_O_2_] independent pathway.

**Fig. 4:**
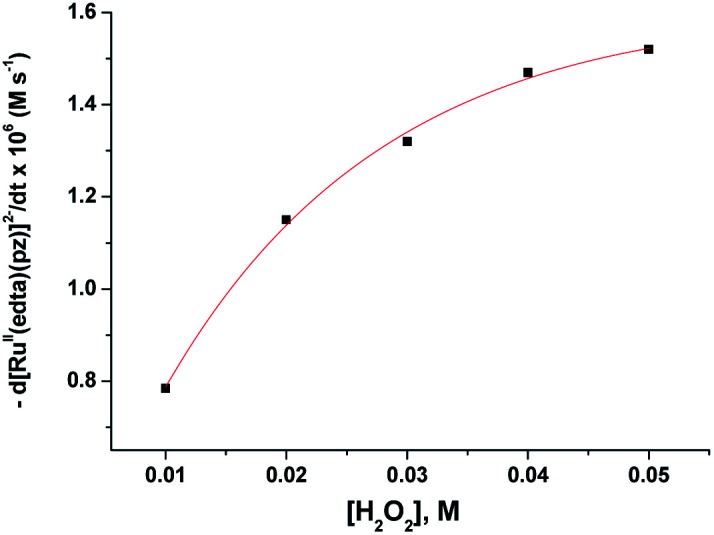
Effect of the concentration of H_2_O_2_ on the maximum rate of the disappearance of [Ru^II^(edta)(pz)]^2−^ with increasing H_2_O_2_ at 25 °C and pH 5.0, [Ru] = 0.25 mM.

The subsequent slower reaction (step II) proceeds in a H_2_O_2_ concentration dependent pathway (Fig. 3b). Typical kinetic traces related to the decrease in absorbance at 462 nm as a function of [H_2_O_2_] at constant pH of 5.0, are shown in Fig. 3b. It is clear that the change in absorbance at 462 nm in each trace is not significant for some initial period of time, however, the length of this lag time shortens with increasing concentration of H_2_O_2_ (Fig. 3b). Nevertheless, the red [Ru^II^(edta)(pz)]^2−^ is not inactive for this apparent dormant period, but rather at a steady-state concentration under the employed conditions. The [Ru^III^(edta)(pz)]^−^ complex (produced *via* oxidation of [Ru^II^(edta)(pz)]^2−^ by H_2_O_2_) is rapidly reduced with ascorbic acid^23^ (present in excess; see S1 in ESI†) to reform the [Ru^II^(edta)(pz)]^2−^ species back in the reaction mixture. Such reaction cycles were continued until the ascorbic acid present in the reacting system is fully consumed. The effect of [H_2_O_2_] on the rate of the disappearance of the [Ru^II^(edta)(pz)]^2−^ complex (estimated from the maximum slope of the absorbance *vs.* time plots given in Fig. 3b), is shown in Fig. 4.

**Scheme 3:**
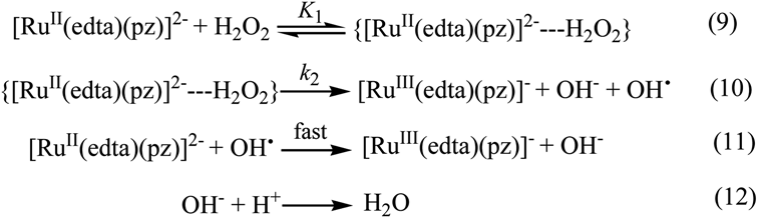
Proposed mechanism for the reduction of H_2_O_2_ by [Ru^II^(edta)(pz)]^2−^.

Based on the above experimental facts, particularly the attainment of a limiting rate at higher [H_2_O_2_] (Fig. 4), the following working mechanism involving a rapid pre-equilibrium, is proposed in Scheme 3 for the reaction of [Ru^II^(edta)(pz)]^2−^ with H_2_O_2_.

In the proposed mechanism (Scheme 3) formation of the {[Ru^II^(edta)(pz)]^2−^ <svg xmlns="http://www.w3.org/2000/svg" version="1.0" width="37.000000pt" height="16.000000pt" viewBox="0 0 37.000000 16.000000" preserveAspectRatio="xMidYMid meet"><metadata> Created by potrace 1.16, written by Peter Selinger 2001-2019 </metadata><g transform="translate(1.000000,15.000000) scale(0.014583,-0.014583)" fill="currentColor" stroke="none"><path d="M80 440 l0 -40 320 0 320 0 0 40 0 40 -320 0 -320 0 0 -40z M880 440 l0 -40 320 0 320 0 0 40 0 40 -320 0 -320 0 0 -40z M1680 440 l0 -40 320 0 320 0 0 40 0 40 -320 0 -320 0 0 -40z"/></g></svg> H_2_O_2_}, an outer-sphere intermediate complex taking place in a pre-equilibrium step (eqn (9)), followed by the rate-determining electron transfer step resulting in the formation of the one-electron oxidized product [Ru^III^(edta)(pz)]^−^ (eqn (10)), accounts for the limiting rate observed in Fig. 4. The OH˙ radical species so formed (eqn (10)), rapidly oxidizes another molecule of [Ru^II^(edta)(pz)]^2−^ to form [Ru^III^(edta)(pz)]^−^ (eqn (11)). The following rate-law (eqn (13) and (14)) can be derived from the reactions in Scheme 3 on the basis that the rate-determining step (eqn (10)) involves the disappearance of the [Ru^II^(edta)(pz)]^2−^ complex monitored at 462 nm.

13 −d[Ru^II^(edta)(pz)]^2−^/d*t* = *k*_2_*K*_1_[Ru]_*t*_[H_2_O_2_]/(1 + *K*_1_[H_2_O_2_])

14 −d*t*/d[Ru^II^(edta)(pz)]^2−^ = 1/*k*_2_[Ru]_*t*_ + 1/*k*_2_*K*_1_[Ru]_*t*_[H_2_O_2_]

A plot of −d*t*/d[Ru^II^(edta)(pz)]^2−^*versus* 1/[H_2_O_2_] was found to be linear (Fig. S5 in ESI†). Considering [Ru]_*t*_ (total concentration of ruthenium) is 0.25 mM, the values of *k*_2_ and *K*_1_ calculated from the intercept and slope of the plot (Fig. S5 in ESI†) are 8.24 × 10^−3^ s^−1^ and 60 M^−1^, respectively, at 25 °C and pH 5.0.

**Fig. 5:**
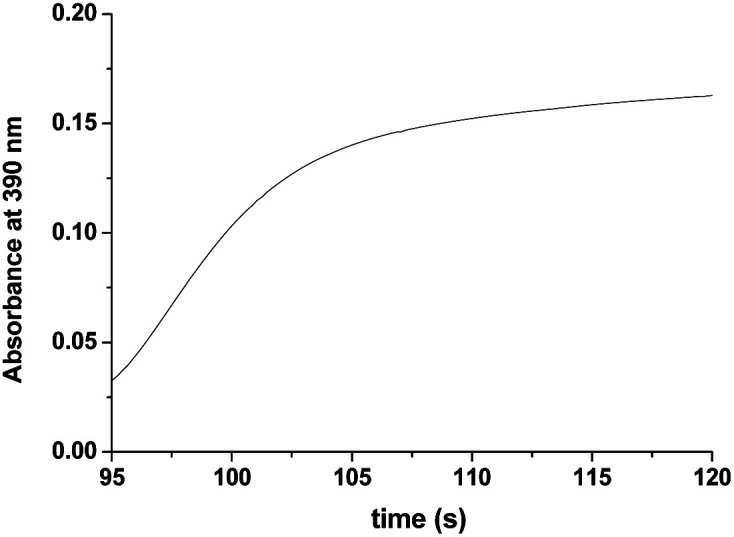
The observed increase in the absorbance at 390 nm with time at higher H_2_O_2_ concentration (step III in Fig. 3b) recorded after 95 s at 25 °C and pH 5.0 (acetate buffer). [Ru] = 0.25 mM [H_2_O_2_] = 40.

As seen in Fig. 3a, after complete disappearance of the peak at 462 nm (attributed to the oxidation of the [Ru^II^(edta)(pz)]^2−^ to [Ru^III^(edta)(pz)]^−^) under the specified conditions (see Fig. 3a), the reaction is followed by a step that involves the formation of a band at 390 nm. This new band is characteristic of the [Ru^V^(edta)O]^−^ complex (*λ*_max_ = 390 nm; *ε*_max_ = 8 × 10^3^ M^−1^ cm^−1^).^24^ The observed increase in the absorbance at 390 nm with time at higher H_2_O_2_ concentration (step III in Fig. 3b), recorded after 95 s delay, is shown separately for clarity in Fig. 5. [Ru^V^(edta)O]^−^ is the product of the oxidation of [Ru^III^(edta)(pz)]^−^ (formed *via* oxidation of the starting [Ru^II^(edta)(pz)]^2−^ (Scheme 3) by H_2_O_2_ remaining present in excess in the reaction mixture).

### Reaction of H_2_O_2_ with [Ru^III^(edta)(pz)]^−^

Above observations, necessitated us to perform further kinetic investigations to understand the mechanistic details pertaining to the formation of [Ru^V^(edta)O]^−^ in the reaction of [Ru^III^(edta)(pz)]^−^ with H_2_O_2_. Noteworthy here, is that the formation of the [Ru^V^(edta)O]^−^ complex through an oxo-transfer reaction from the precursor oxidant ROOH (ROOH = H_2_O_2_, ^*t*^BuOOH and KHSO_5_) to [Ru^III^(edta)(H_2_O)]^−^ was reported by us.^25–27^ An inner-sphere mechanism involving the formation of the [(edta)Ru^III^(OOR)]^2−/3−^ intermediates (R = H, ^*t*^Bu and SO_3_^−^) in a rapid pre-equilibrium step, followed by the rate-controlling heterolytic cleavage of the O–O bond to produce the [Ru^V^(edta)O]^−^ complex, was proposed.^25–27^

**Fig. 6:**
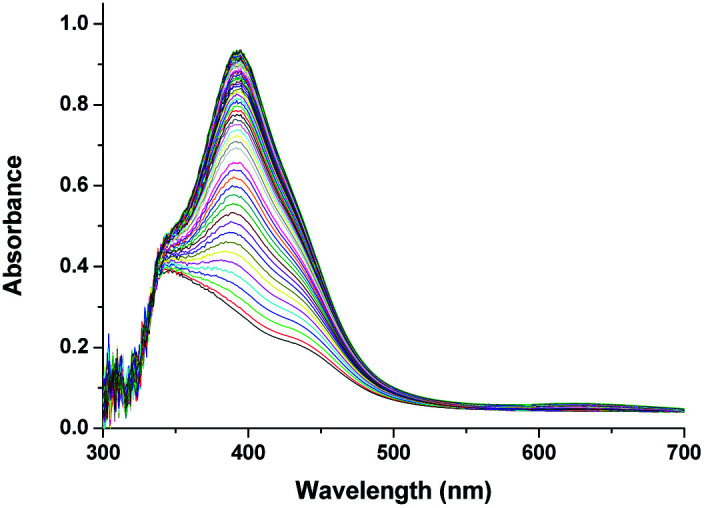
Spectral changes that occurred during oxidation of [Ru^III^(edta)(pz)]^−^ with H_2_O_2_ at 25 °C and pH 5.0, [Ru] = 0.25 mM, [H_2_O_2_] = 0.4 mM.

**Fig. 7:**
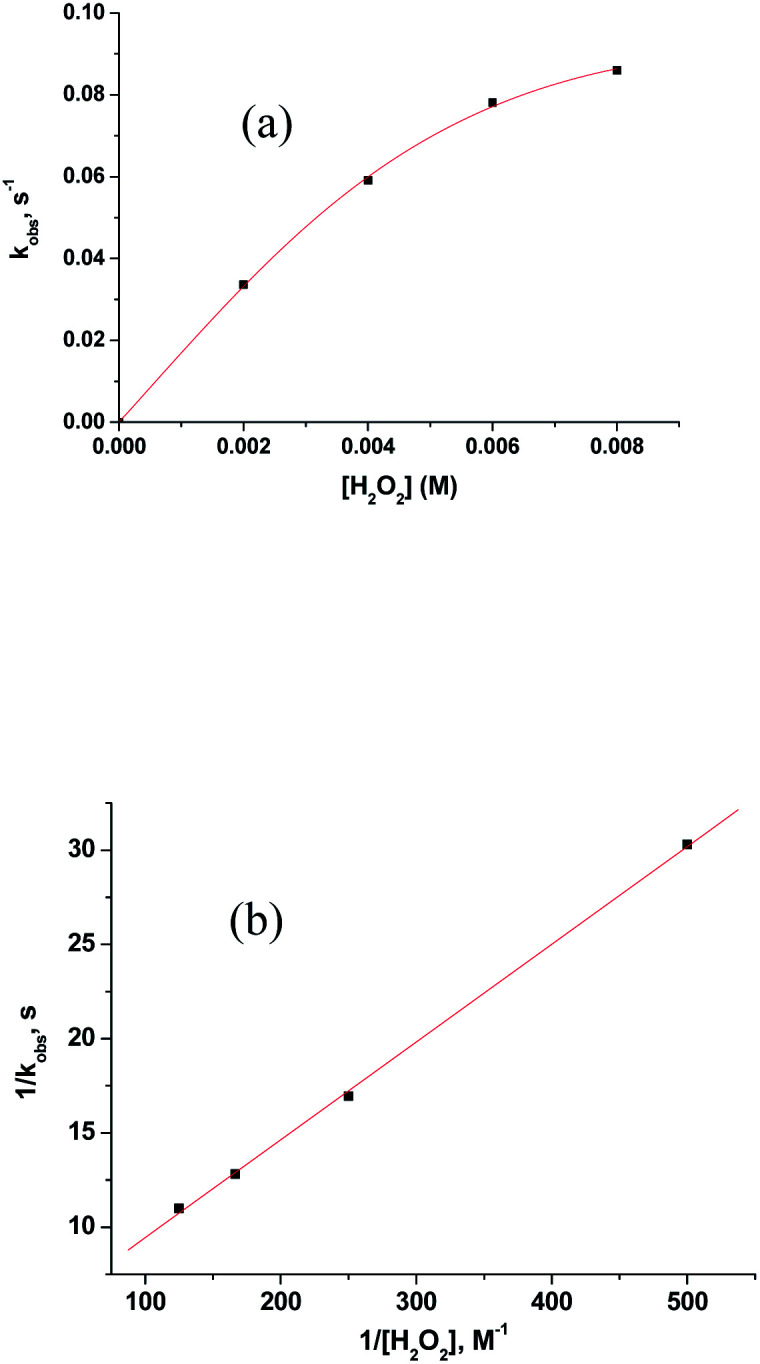
(a) Effect of the concentration of H_2_O_2_ on the *k*_obs_ values for the reaction of [Ru^III^(edta)(pz)]^−^ with H_2_O_2_ at 25 °C and pH 5.0. [Ru] = 0.2 mM. (b) Plot of 1/*k*_obs_*versus* 1/[H_2_O_2_].

**Scheme 4:**
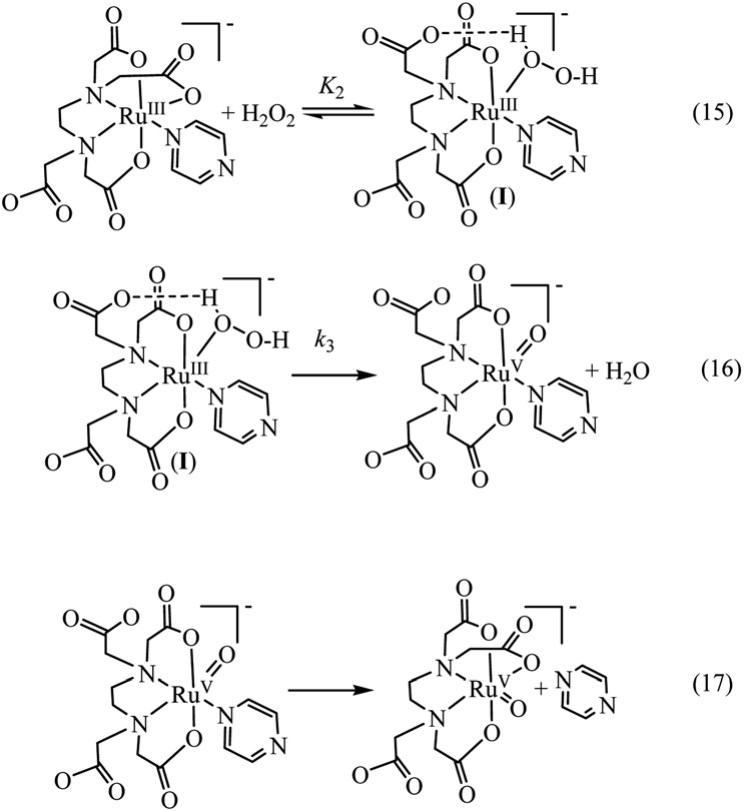
Proposed mechanism for the reduction of H_2_O_2_ by [Ru^III^(edta)(pz)]^−^.

In Fig. 6 the UV-vis spectral changes with time that occurred upon mixing aqueous solutions of [Ru^III^(edta)(pz)]^−^ and H_2_O_2_, are shown. The observed spectral changes (Fig. 6) are attributed to the oxidation of [Ru^III^(edta)(pz)]^−^ to [Ru^V^(edta)O]^−^ under the specified conditions. The kinetic traces at 390 nm, generated from the recorded spectra, are presented in Fig. S6 (see ESI†). The effect of the H_2_O_2_ concentration on the values of the observed rate constant (*k*_obs_) is shown in Fig. 7a. The observed saturation of the *k*_obs_ values at higher H_2_O_2_ concentration, suggests a process that involves a rate-limiting pre-equilibrium step followed by the rate-determining formation of [Ru^V^(edta)O]^−^ species. On the basis of a pre-equilibrium approach, the above kinetic results for the formation of [Ru^V^(edta)O]^−^ in the reaction of [Ru^III^(edta)(pz)]^−^ with H_2_O_2_, can be accounted for in terms of the following mechanism proposed in Scheme 4.

Considering H_2_O_2_ being a poor nucleophile that cannot efficiently replace pyrazine (a strong aromatic N-heterocyclic π-acidic ligand) to form [Ru^III^(edta)(OOH)]^2−^ species (as observed in case of the reaction of [Ru^III^(edta)(H_2_O)]^−^ with H_2_O_2_),^27^ we invoke the formation of a [Ru^III^(edta)(H_2_O_2_)(pz)]^−^ intermediate *via* coordination of H_2_O_2_ to the metal centre by dislodging the coordinated acetate arm of the ‘edta’ ligand in the pre-equilibrium step (eqn (15)). The dangling acetate arm plausibly stabilizes the proposed intermediate species by forming a transient hydrogen bond between carboxylate oxygen atom of the dangling acetate arm and H-atom of the H_2_O_2_ coordinated to the metal centre as shown pictorially in Scheme 4 (eqn (15)). However, no spectral evidence in favour of the formation of the proposed intermediate was observed. Noteworthy here is that a distinct spectral evidence (formation of a shoulder in the 390 nm band at 425 nm) confirming the formation of [Ru^III^(edta)(OOH)]^2−^ intermediate species was observed during the course of the reaction of [Ru^III^(edta)(H_2_O)]^−^ with H_2_O_2_.^27^ In the subsequent rate-determining step, the [Ru^III^(edta)(H_2_O_2_)(pz)]^−^ intermediate undergoes heterolytic cleavage of the O–O bond to produce [Ru^V^(edta)(pz)O]^−^ species, with concomitant release of a water molecule (eqn (16)) in a concerted pathway. However, the metal–pyrazine bond at a higher oxidation state of the metal in [Ru^V^(edta)(pz)O]^−^ becomes less stable, and the π-acidic ligand pyrazine, thereby dissociates allowing the dangling carboxylate group to bind to the metal centre again to produce the [Ru^V^(edta)O]^−^ product complex in a kinetically inconsequential step (eqn (17)). The following rate-law (eqn (18) and (19)) can be derived for the reactions in Scheme 4 on the basis of the rate-determining formation of the [Ru^V^(edta)O]^−^ complex.

18 *k*_obs_ = *k*_3_*K*_2_[H_2_O_2_]/{1 + *K*_2_[H_2_O_2_]}

19 1/*k*_obs_ = 1/*k*_3_ + 1/*k*_3_*K*_2_[H_2_O_2_]

The plot of 1/*k*_obs_*versus* 1/[H_2_O_2_] is linear (Fig. 7b), and the values of *k*_3_ and *K*_2_ from the intercept and slope are 0.23 ± 0.03 s^−1^ and 82 ± 2 M^−1^, respectively, at 25 °C and pH 5. The product *k*_3_*K*_2_ presenting the over-all second-order rate constant for the formation of [Ru^V^(edta)O]^−^ has a value of 18.8 M^−1^ s^−1^ (estimated by using the values of *k*_3_ and *K*_2_ stated above) is just little more than half of the value (33 M^−1^ s^−1^) reported for the reaction of [Ru^III^(edta)(H_2_O)]^−^ with H_2_O_2_.^27^

The above findings taken together with that reported for the reaction of [Ru^III^(edta)(H_2_O)]^−^ with H_2_O_2_,^27^ are suggestive of the fact that the reduction of H_2_O_2_ by both [Ru^III^(edta)(pz)]^−^ and [Ru^III^(edta)(H_2_O)]^−^ resulting in the formation of [Ru^V^(edta)O]^−^ and H_2_O as ultimate reaction products, proceeds through an inner-sphere pathway demonstrating similar kinetic features. Although the common suggested mechanism involves heterolytic cleavage of the O–O bond, the ability of H_2_O_2_ to bind to the Ru(iii)-centre through a ligand substitution process, governs the efficiency of the H_2_O_2_ reduction process. In this regard, [Ru^III^(edta)(H_2_O)]^−^ due to its unusual lability towards a substitution reaction, has an advantage over [Ru^III^(edta)(pz)]^−^.

### Reaction of O_2_ and H_2_O_2_ with [Ru^III^(edta)(H_2_O)]^−^

Noteworthy here, is that the [Ru^III^(edta)(H_2_O)]^−^ complex also exhibited a metal based one-electron transfer reaction, and the *E*_1/2_ value reported for the [Ru^III^(edta)(H_2_O)]^−^/[Ru^II^(edta)(H_2_O)]^2−^ is −0.018 V (*vs.* SHE),^19^ which is much more negative than that reported for the [Ru^III^(edta)(pz)]^−^/[Ru^II^(edta)(pz)]^2−^ couple (*E*_1/2_ = 0.252 V *vs.* SHE).^19^

**Fig. 8:**
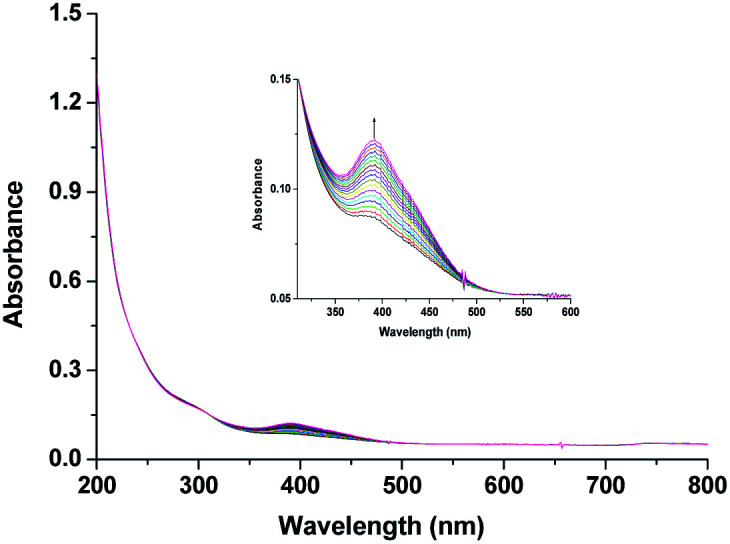
Spectral changes that occurred during the reaction of [Ru^II^(edta)(H_2_O)]^2−^ with O_2_ at 25 °C and pH 5.0. Inset. Exaggerated spectral changes pertaining to the formation of [Ru^V^(edta)O]^−^. [Ru] = 0.025 mM, [O_2_] = 0.5 mM.

**Scheme 5:**
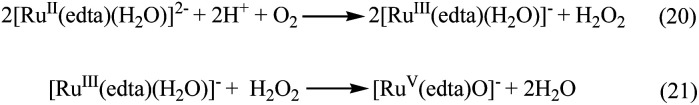
Proposed mechanism for the reaction of [Ru^II^(edta)(H_2_O)]^2−^ with O_2_.

Though [Ru^II^(edta)(H_2_O)]^2−^ is a stronger reductant thermodynamically than [Ru^II^(edta)(pz)]^2−^ towards O_2_ reduction, detailed kinetic investigations were practically not feasible because of very insignificant spectral differences between aqua-analogues of Ru(iii) and Ru(ii)–edta complexes. The [Ru^III^(edta)(H_2_O)]^−^ in water is almost featureless over the entire visible range of the spectrum, but exhibits a strong absorption band at 280 nm (*ε*_max_ = 2800 ± 50 M^−1^ cm^−1^) and a shoulder at 350 nm (*ε*_max_ = 680 ± 60 M^−1^ cm^−1^) in the UV region.^19^ The Ru(ii) analogue, [Ru^II^(edta)(H_2_O)]^2−^ displays almost similar spectral features exhibiting an intense band at 282 nm (*ε*_max_ = 2900 ± 100 M^−1^ cm^−1^) and a weak shoulder at 427 nm (*ε*_max_ = 260 ± 15 M^−1^ cm^−1^).^19^ Nevertheless, the formation of the [Ru^V^(edta)O]^−^ complex was evidenced in the spectral changes (Fig. 8) that occurred in the reaction of [Ru^II^(edta)(H_2_O)]^2−^ with oxygen. Time resolved spectral changes recorded (Fig. 8) after mixing of the aqueous solution of [Ru^II^(edta)(H_2_O)]^2−^ (0.025 mM) with an oxygen saturated aqueous solution, clearly revealed the gradual build-up of the peak at 390 nm (inset of Fig. 8) characteristic for the [Ru^V^(edta)O]^−^ complex.^24^ The above spectral observations may be explicable in terms of the following reactions as outlined in Scheme 5.

In the above proposed mechanism (admittedly speculative), the reduction of O_2_ to H_2_O_2_ with concomitant formation of the [Ru^III^(edta)(H_2_O)]^−^ takes place in an outer-sphere pathway (eqn (20)). The appearance of the peak at 390 nm in Fig. 8 can be accounted for by the reaction of H_2_O_2_ with the one-electron oxidized product [Ru^III^(edta)(H_2_O)]^−^ (eqn (21)).

**Fig. 9:**
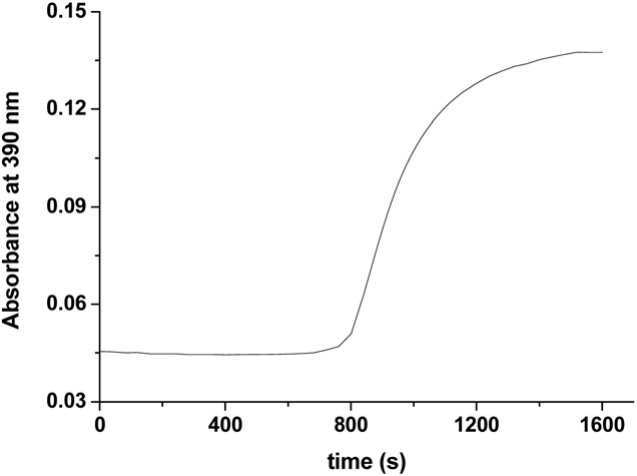
Absorbance *vs.* time trace recorded at 25 °C for the reaction of [Ru^II^(edta)(H_2_O)]^2−^ with H_2_O_2_ at pH 5.0, [Ru] = 0.025 mM [H_2_O_2_] = 10 mM.

**Scheme 6:**
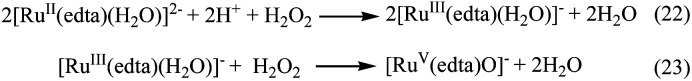
Proposed mechanism for the reaction of [Ru^II^(edta)(H_2_O)]^2−^ with H_2_O_2_.

Formation of [Ru^V^(edta)O]^−^ was also noticed in the direct reaction of [Ru^II^(edta)(H_2_O)]^2−^ with H_2_O_2_. The absorbance *versus* time profile (recorded at 390 nm), pertaining to the formation of [Ru^V^(edta)O]^−^ in the reaction of [Ru^II^(edta)(H_2_O)]^2−^ with H_2_O_2_, is given in Fig. 9. As it is seen, the kinetic trace exhibits a clear initial induction period. Above observations may be explicable in terms of the following reaction scheme (Scheme 6) proposed for the reduction of H_2_O_2_ by [Ru^II^(edta)(H_2_O)]^2−^.

**Fig. 10:**
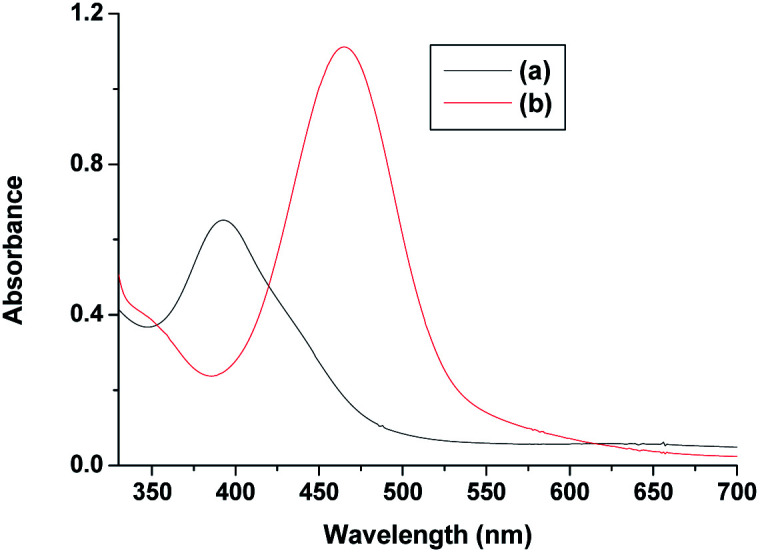
Spectra of (a) [Ru^V^(edta)O]^−^ and (b) after addition of ascorbic acid and pyrazine to the solution of [Ru^V^(edta)O]^−^ at 25 °C and pH 5.0.

The observed initial induction period (Fig. 9) is explicable in terms of a catalytic cycle wherein the [Ru^III^(edta)(H_2_O)]^−^ complex formed concomitantly during reduction of H_2_O_2_ by [Ru^II^(edta)(H_2_O)]^2−^ (eqn (22)) rapidly undergoes reduction by the ascorbic acid present in excess in the reacting system (see S1 in ESI†), regenerating the [Ru^II^(edta)(H_2_O)]^2−^ species in the reaction mixture. Such reaction cycles sustained until the ascorbic acid present in the reacting system are completely exhausted. At the end of the above referred catalytic process, formation of [Ru^V^(edta)O]^−^ takes place in the reaction of [Ru^III^(edta)(H_2_O)]^−^ with H_2_O_2_ (eqn 23). Noteworthy here, is that the sequential addition of a fresh amount of ascorbic acid and pyrazine to the solution obtained just after completion of the [Ru^V^(edta)O]^−^ formation, immediately recovered the spectral features of [Ru^II^(edta)(pz)]^2−^ (Fig. 10), confirming that the precursor [Ru^II^(edta)(H_2_O)]^2−^ complex is not degraded during the aforesaid redox processes under the specified experimental conditions.

**Scheme 7:**
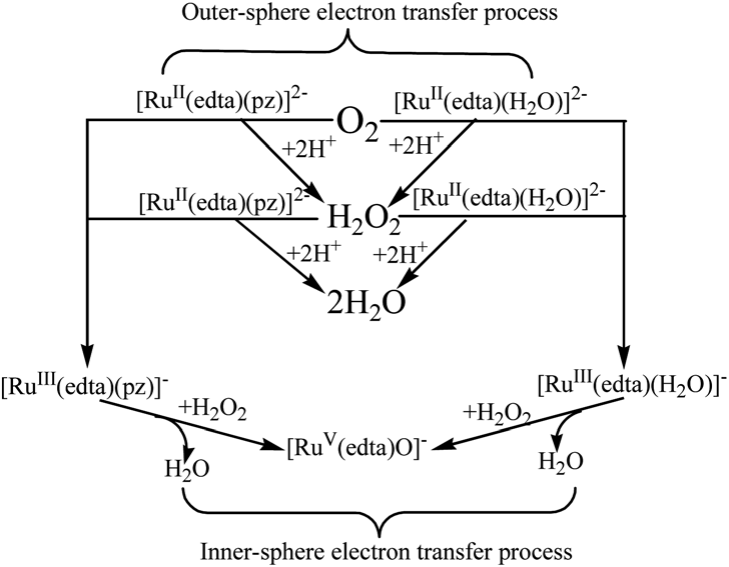
Schematic presentation of O_2_ and H_2_O_2_ reduction by [Ru^II/III^(edta)(pz)]^2−/−^ and [Ru^II/III^(edta)(H_2_O)]^2−/−^ in aqueous solution.

In summary, a close examination of the mechanistic pathways proposes in Schemes 1–5, coupled with our mechanistic understanding of the reaction of [Ru^III^(edta)(H_2_O)]^−^ with H_2_O_2_, strongly suggests that both the [Ru^II^(edta)(H_2_O)]^2−^ and [Ru^II^(edta)(pz)]^2−^ could affect sequential reduction of O_2_ to H_2_O_2_ and further to H_2_O in an outer-sphere electron transfer pathway as depicted in Scheme 7. The key-feature is that both the [Ru^III^(edta)(pz)]^−^ and [Ru^III^(edta)(H_2_O)]^−^ complexes (generated by one-electron oxidation of their respective Ru(ii)-analogues) are found effective in reducing H_2_O_2_ to H_2_O, however, through an inner-sphere electron transfer pathway (Scheme 7).

### [Ru^III^(edta)(pz)]^−^ mediated electrochemical reduction of O_2_

**Fig. 11:**
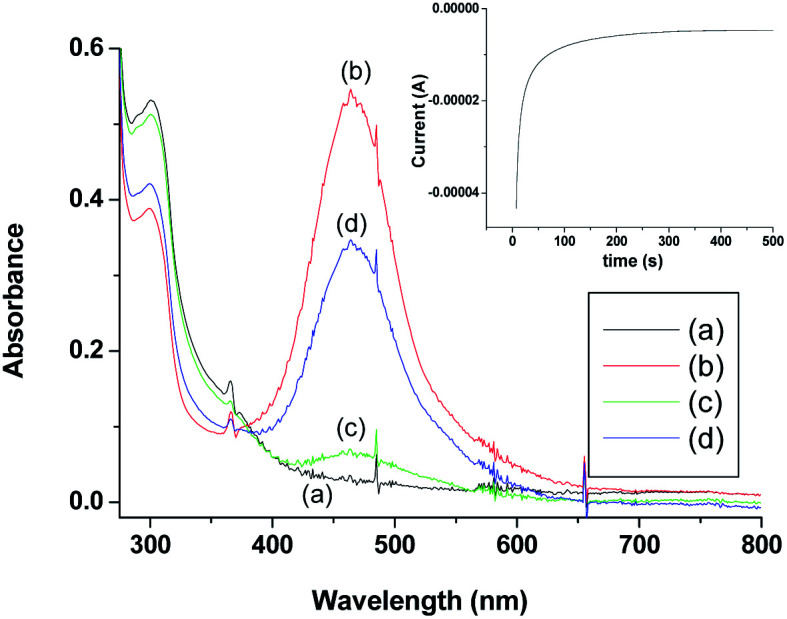
Spectra of (a) solution of [Ru^III^(edta)(pz)]^−^ in acetate buffer prior to the electrolysis (solution-1); (b) after electrolysis of solution-1 at −0.05 V (*vs.* Ag/AgCl); (c) after switching off the potential followed by the oxygenation of the electrolysed solution (for 300 s) and (d) after second run of electrolysis of the oxygenated solution. [Ru^III^] = 0.5 mM, pH = 5.0.

Being inspired by the aforementioned results, we performed a brief spectro-electrochemical experimentation (see S2 in ESI†), the results of which evidently reveal that [Ru^III^(edta)(pz)]^−^ could act as a ‘molecular redox catalyst’ for electrochemical reduction of O_2_. Reduction of [Ru^III^(edta)(pz)]^−^ to [Ru^II^(edta)(pz)]^2−^ was achieved electrochemically by carrying out constant potential electrolysis (at −0.05 V *vs.* Ag/AgCl) of the solution of [Ru^III^(edta)(pz)]^−^ (in acetate buffer solution at pH 5.0). Spectrum of the solution of [Ru^III^(edta)(pz)]^−^ (0.5 mM in acetate buffer) is shown in Fig. 11a. Constant potential electrolysis of the solution of [Ru^III^(edta)(pz)]^−^ leading to the formation of the [Ru^II^(edta)(pz)]^2−^ species was evident spectrophotometrically by the appearance of its characteristic peak at 462 nm (Fig. 11b). The typical current *versus* time plot pertaining to the above mentioned electrochemical process is shown in the inset of the Fig. 11. After withdrawal of the potential, O_2_ was bubbled through the electrolysed solution for 300 s. The spectral changes thereafter (Fig. 11c) show appreciable collapse of the peak at 462 nm, which is consistent with the re-oxidation of [Ru^II^(edta)(pz)]^2−^ to [Ru^III^(edta)(pz)]^−^ by dioxygen (O_2_). Electrolysis of the solution (at −0.05 V) for 500 s again regenerates the [Ru^II^(edta)(pz)]^2−^ species as evident spectrophotometrically (Fig. 11d). However, the intensity of the band at 462 nm as noticed in the spectrum (Fig. 11d) is significantly smaller in comparison to that observed in the spectrum (Fig. 11b). This may plausibly be associated with the fact that the unconsumed dissolved oxygen present in the reacting system prior to the second run of electrolysis, may compete with the electrochemical reduction process for which complete formation of the [Ru^II^(edta)(pz)]^2−^ could not take place within the time period the voltage was on (500 s). Observation of the band (at 462 nm), with a higher absorbance in the spectrum of the electrolysed solution, which was deoxygenated through argon purging prior to the electrolysis (see Fig. S7 in ESI†), supports our above argument.

**Scheme 8:**
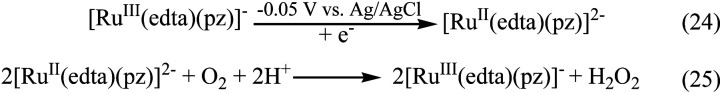
Schematic presentation of [Ru^III^(edta)(pz)]^−^ mediated electrochemical reduction of O_2_ in aqueous solution.

Based on the above observations, taken together with that reported for the reaction of [Ru^II^(edta)(pz)]^2−^ with molecular oxygen (O_2_) in the preceding section, the role of [Ru^III^(edta)(pz)]^−^ as an electron transfer redox catalyst in the electrochemical reduction of O_2_ may be outlined in Scheme 8.

## Conclusions

In conclusion, the results of the present study reveal that in the overall reactions, whether chemical or electrochemical, dioxygen (O_2_) is reduced to hydrogen peroxide (H_2_O_2_) *via* electron transfer reaction, and the [Ru^III^(edta)(pz)]^−^ complex acts as a redox relay for electron transmission. In case of the chemical process, it takes electrons from ascorbic acid to reduce O_2_ to H_2_O_2_, whereas in case of the electrochemical process, it uses the electrons from the working electrode to effect electrochemical reduction of O_2_ to H_2_O_2_ in aqueous acidic solution. The results of our studies further ascertain that both the [Ru^II^(edta)(pz)]^2−^ and [Ru^II^(edta)(H_2_O)]^2−^ complexes in presence of electron donors can reduce O_2_ to H_2_O_2_ and H_2_O_2_ to H_2_O efficiently in a sequential manner. The redox mediating properties of the aforesaid Ru(edta) complexes along with their wide range of chemically accessible oxidation states (II to V), and their durability in the redox processes, are indeed intriguing and prospective. Our results may shed light towards mechanistic understanding of the homogeneously catalysed reduction of O_2_ and H_2_O_2_ and provide pointers for future research pertaining to the application of such metal complexes in the field of fuel cells and metal–air batteries.

## Conflicts of interest

There are no conflicts to declare.

## Supplementary Material

RA-011-D1RA03293C-s001
