# Supplementary material for: RuIII(edta) complexes as molecular redox catalysts in chemical and electrochemical reduction of dioxygen and hydrogen peroxide: inner-sphere versus outer-sphere mechanism
Source: RSC Adv. 2021 Jun 16;11(35):21359–66. doi: 10.1039/d1ra03293c (PMC9034107; doi:10.1039/d1ra03293c)
Supplement: RA-011-D1RA03293C-s001 [file RA-011-D1RA03293C-s001.pdf]

Electronic Supplementary Material (ESI) for RSC Advances.
This journal is © The Royal Society of Chemistry 2021

ESI

Ru^{III}(edta) complexes as molecular redox catalyst in chemical and electrochemical reduction of dioxygen and hydrogen peroxide: Inner-sphere versus outer-sphere mechanism†

Debabrata Chatterjee,*[a,b] Marta Chrzanowska,[b] Anna Katafias,[b] Maria Oszajca,[c] and Rudi van Eldik*[b,c,d]

[a)] Vice-Chancellor's Research Group at Zoology Department, University of Burdwan, Burdwan-713104, India, [b)] Faculty of Chemistry, Nicolaus Copernicus University in Toruń, Gagarina 7, 87-100 Toruń, Poland, [c)] Faculty of Chemistry, Jagiellonian University, Gronostajowa 2, 30-387 Kraków, Poland and [d)]Department of Chemistry and Pharmacy, University of Erlangen-Nuremberg, Egerlandstr. 1, 91058 Erlangen, Germany.

S1 Freshly prepared solutions of [Ru^{II}(edta)(pz)]$^{2-}$ were always used throughout all experiments. For this purpose, deaerated solutions of [Ru^{III}(edta((pz)]$^{-}$ of desired concentration, were reduced by using a slight excess of ascorbic acid ([RuIII]: [ascorbic acid] = 1:5). The pH of the reacting solution was maintained at 5.0 by using NaOH/HCl solution. The whole process was carried out strictly under argon atmosphere. The same procedure was adapted for preparation of its aqua-analogue, [Ru^{II}(edta)(H$_2$O)]$^{2-}$.

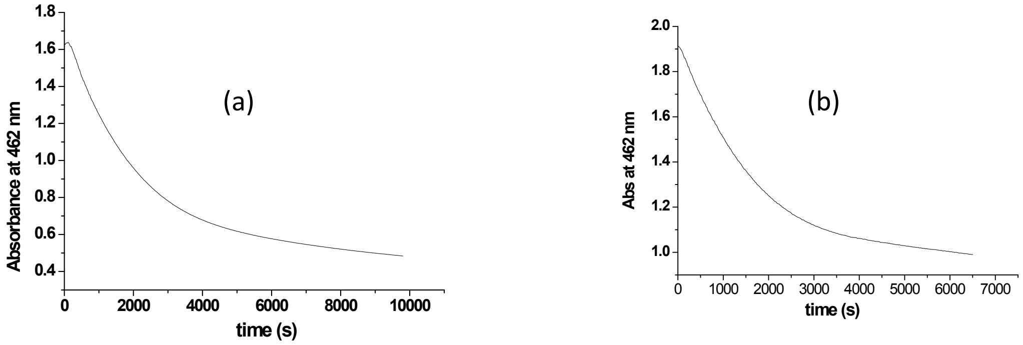

Fig. S1 Kinetic traces recorded at 462 nm for the reaction of [Ru^{II}(edta)(pz)]$^{2-}$ with O_2 at 25 °C and pH 5.0. [O_2] = (a) 0.125 and (b) 0.25 mM. [Ru] = 0.25 mM.

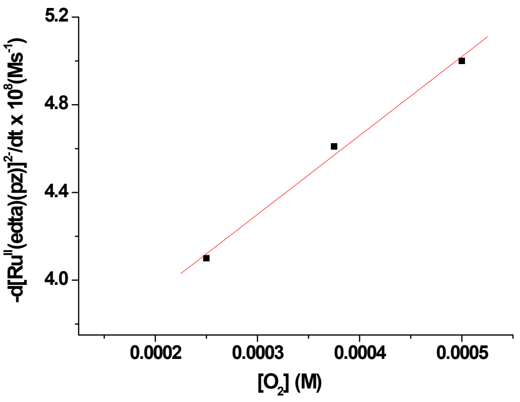

Fig. S2 Plot of initial rate $-d[Ru^{II}(edta)(pz)^{2-}]/dt$ versus $[O_2]$ at 25 °C and pH 5.0 (acetate buffer). [Ru] = 0.25 mM.

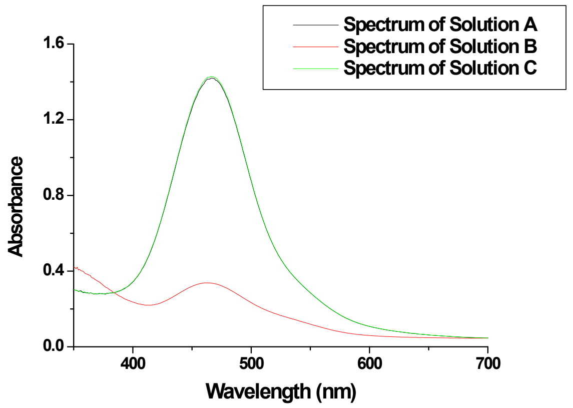

Fig. S3 Spectra of solution A (0.2 mM deaerated solution of $[Ru^{II}(edta)(pz)]^{2-}$), solution B (obtained after reaction with O_2), and solution C (obtained after addition of fresh ascorbic acid to solution B).

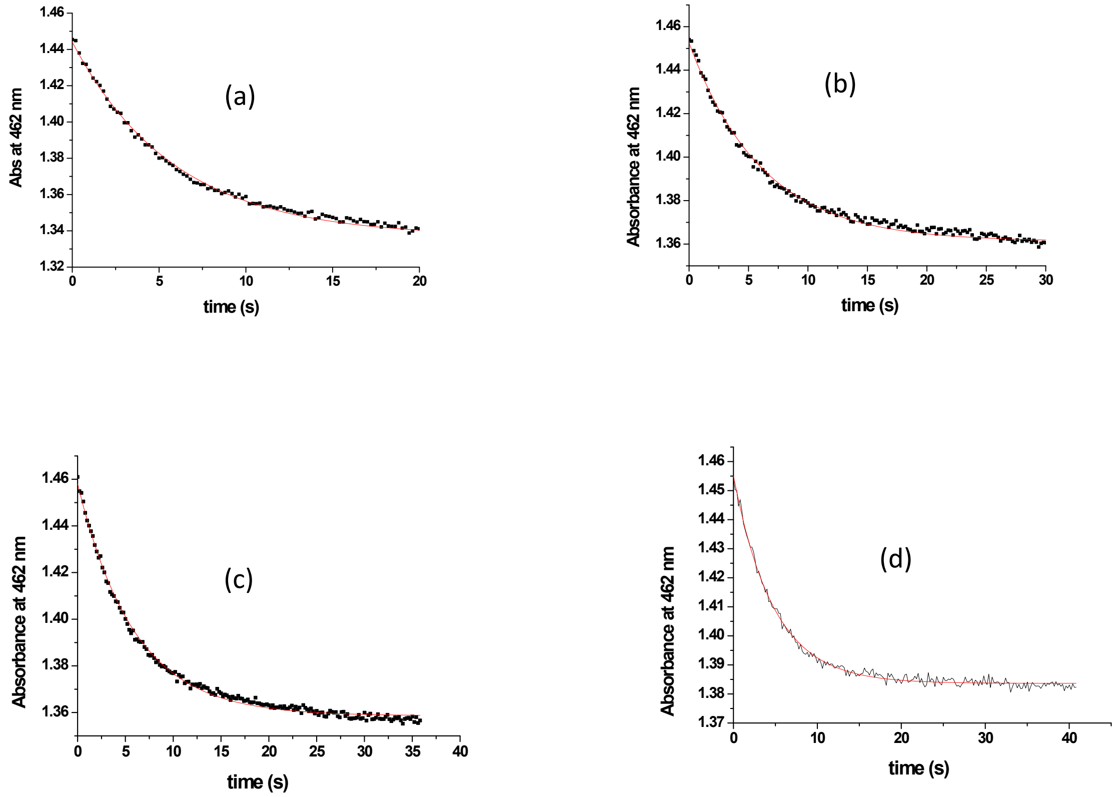

Fig. S4 Kinetic traces (recorded at 462 nm) corresponding to the first-step (I) of the reaction of $[Ru^{II}(edta)(pz)]^{2-}$ with H_2O_2 at 25 °C and pH 5.0 (acetate buffer). [Ru] = 0.25 mM, $[H_2O_2]$ = (a) 10 mM, (b) 20 mM, (c) 30 mM and (d) 40 mM.

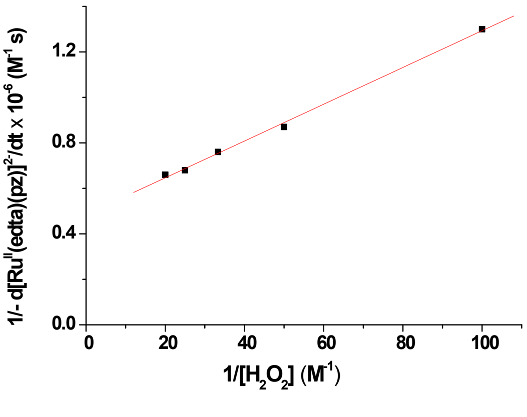

Fig. S5 Plot of 1/rate vs. $1/[H_2O_2]$ for reaction of $[Ru^{II}(edta)(pz)]^{2-}$ with H_2O_2 at 25 °C and pH 5.0 (acetate buffer), [Ru] = 0.25 mM.

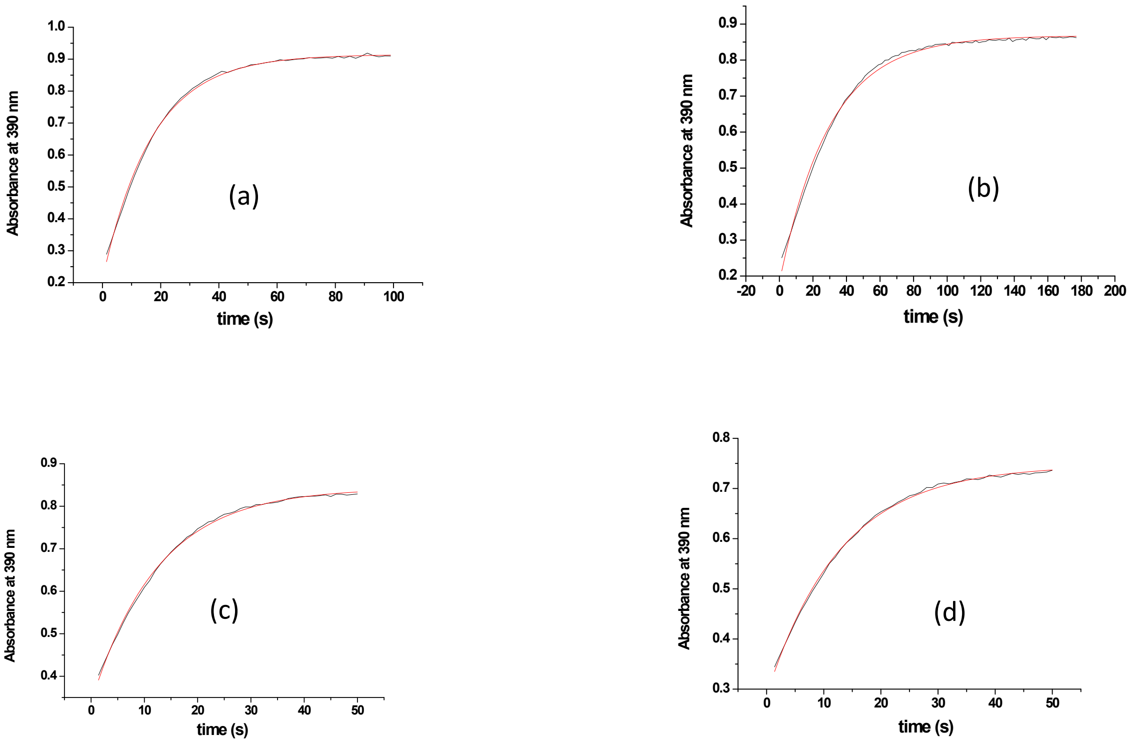

Fig. S6 Kinetic traces recorded at 390 nm for the reaction of $[Ru^{III}(edta)(pz)]^-$ with H_2O_2 at 25 °C and pH 5.0 (acetate buffer). $[H_2O_2]$ = (a) 2 mM, (b) 4 mM, (c) 6 mM and (d) 8 mM. [Ru] = 0.2mM.

S2 Spectro-electrochemical measurements were carried out using a Potentiostat (Autolab) in parallel with the diode array spectrophotometer (Hewlett-Packard). The three electrode system was designed for a rectangular quartz cell having 1 mm internal path length. A platinum gauze as working electrode, platinum wire as auxiliary electrode and Ag/AgCl reference electrode were used for achieving constant potential electrolysis. Solution of $[Ru^{III}(edta)(pz)]^-$ was prepared by mixing an equal volume of the solution of $[Ru^{III}(edta)(H_2O)]^-$ (1 mM) with an equal volume of the pyrazine solution (6.5 mM) in acetate buffer (pH 5.0).

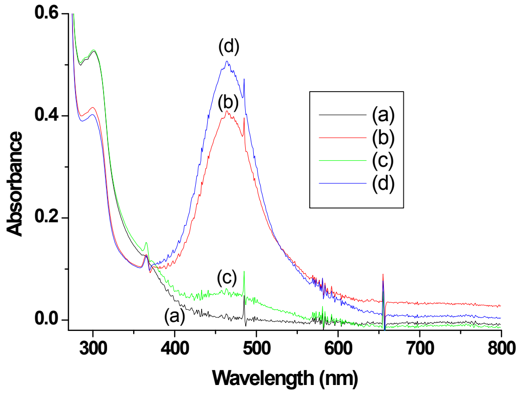

Fig. S7 Spectra of (a) solution of $[Ru^{III}(edta)(pz)]^{-}$ in acetate buffer prior to the electrolysis (Solution-1); (b) after electrolysis (1st run) of Solution-1 at -0.05 V (vs Ag/AgCl); (c) after switching off the potential followed by the oxygenation of the electrolysed solution and (d) after electrolysis (2nd run) of the deoxygenated solution (through argon purging). $[Ru^{III}]$ = 0.5 mM, pH 5.0.
